# Gadobutrol Precedes Gd-DTPA in Abdominal Contrast-Enhanced MRA and MRI: A Prospective, Multicenter, Intraindividual Study

**DOI:** 10.1155/2019/9738464

**Published:** 2019-12-02

**Authors:** Xijiao Liu, Zhengyan Li, Weiwei Zhang, Caiwei Yang, Yike Diao, Ting Duan, Yu Fu, Jing Ren, Song Bin

**Affiliations:** ^1^Department of Radiology, West China Hospital, Sichuan University, Chengdu, Sichuan Province, China; ^2^Department of Radiology, The First Hospital of Jilin University, Changchun, Jilin Province, China; ^3^Department of Radiology, Sichuan Cancer Hospital, Chengdu, Sichuan Province, China

## Abstract

**Objective:**

To qualitatively and quantitatively compare the contrast-enhanced magnetic resonance angiography (MRA) and magnetic resonance imaging (MRI) in one-stop shop of abdominal imaging with Gadobutrol and Gd-DTPA at equimolar doses of gadolinium.

**Materials and Methods:**

This was a prospective designed, multiple center, intraindividual comparison study. All volunteers underwent Gadobutrol- and Gd-DTPA-enhanced MRA and MRI in one-stop shop. Qualitative analysis for large vessels and small vessels was performed by a three-point scale, while for minute small vessels, by a five-point scale. Quantitative analysis was performed for large vessels by signal-to-noise ratio (SNR) and contrast-to-noise ratio (CNR). Visceral organ enhancements on the equilibrium phase were also analyzed. Wilcoxon matched-pair signed-rank tests were used to evaluate the qualitative and quantitative results.

**Results:**

40 volunteers were enrolled. Qualitative analyses results for large vessels, small vessels, and minute small vessels of Gadobutrol and Gd-DTPA were 20.98 ± 2.11, 6.03 ± 1.03, and 3.41 ± 1.18 and 20.01 ± 2.18, 5.28 ± 1.67, and 2.61 ± 1.40, respectively. Wilcoxon signed-rank tests revealed Gadobutrol-enhanced MRA was superior to that of Gd-DTPA significantly for small vessels (*p*=0.028) and minute small vessels (*p*=0.007). For quantitative analysis of large vessels, no statistic difference was found. Gadobutrol-enhanced MRI had higher CNR of the liver (*p*=0.003), spleen (*p*=0.001), and pancreas (*p*=0.001) and higher SNR of spleen (*p*=0.009) than those of Gd-DTPA statistically.

**Conclusion:**

Our study proved Gadobutrol was superior to Gd-DTPA in qualitative analysis of CE-MRA and quantitative analysis of visceral organ enhancement on CE-MRI in abdomen of healthy volunteers. Gadobutrol may be more suitable for abdominal one-stop examination for CE-MRA and CE-MRI.

## 1. Introduction

The U.S. Food and Drug Administration approved the first gadolinium-based contrast agent (GBCA) in 1988. Ever since then, contrast-enhanced magnetic resonance angiography (CE-MRA) and contrast-enhanced magnetic resonance imaging (CE-MRI) have become used in medicine. They have high soft tissue resolution, as well as improved reproducibility. Nowadays, their crucial role in diagnostic and follow-up imaging of pathological lesions is widely recognized.

Most GBCAs in clinics are 0.5 M Gd chelate, for example, gadopentetate dimeglumine (Gd-DTPA, Magnevist, Bayer Schering, Berlin, Germany) and Gd-DTPA-BMA (Omniscan) [1]. Gd-DTPA is an ionic and linear GBCA (longitudinal relaxation time (T1) relaxivity of 4.1 L/mmol/s at 1.5 Tesla, 37°C in human plasma). Gadobutrol is a 1.0 molar macrocyclic GBCA with low osmolarity and viscosity. The higher concentration of Gadobutrol results in high relaxivity (T1 relaxivity of 5.3 L/mmol/s at 1.5 Tesla, 37°C in human plasma) and high signal intensity on a T1-weighted image [2, 3]. It has been speculated that the high Gd concentration and halved injection volume of Gadobutrol facilitate a sharper bolus peak and yield a higher intravascular concentration during the MRA acquisition, which would also have advantages in dynamic imaging [4]. Previous studies report Gadobutrol preceding delineation of small vessels with comparatively increased signal-to-noise ratio (SNR) and contrast-to-noise ratio (CNR) than Gd-DTPA in the vascular territory [5–7], while contradictory results showed there was no statistic difference between the two contrast agents for CE-MRA [8].

With recent developments in contrast agents and pulse sequences, some state-of-the-art techniques greatly improve the MR image quality by overcoming the time and spatial resolution limitations [9]. A comprehensive MR imaging protocol may be used to assess the arteries and parenchyma enhancement simultaneously, called “one-stop.” This technique could avoid repeated injection of the contrast agent for twice examination and make reasonable use of medical resonance. Previous studies determined the suitability and feasibility of a “one-stop” in the abdomen [10, 11]. So far, there is little literature on Gadobutrol or Gd-DTPA in abdominal one-stop shop imaging for CE-MRA and CE-MRI.

The purpose of this study was to innovatively determine whether Gadobutrol-enhanced MRA and MRI demonstrating qualitatively and quantitatively superior to Gd-DTPA-enhanced MRA and MRI at equimolar doses of gadolinium in abdominal “one-stop” imaging.

## 2. Materials and Methods

This prospective designed, blinded, intraindividual comparison study was approved by the institutional review boards, and the study was accomplished in three medical centers.

### 2.1. Study Population

The health volunteers met the inclusions: 18–75 years old; without any history of vascular or renal disease or liver disease; normal renal function; and voluntarily signed the informed consent form. The exclusion criteria were as follows: pregnancy or lactation; contraindications to MRI examination such as claustrophobia; contraindication to use of GBCAs (including suspected or confirmed subjects); use of any contrast agent within 72 hours prior to the examination; and risk of clinical deterioration that could have an adverse impact on participation.

### 2.2. MR Imaging and Study Protocol

All volunteers underwent both Gadobutrol- and Gd-DTPA-enhanced MRA and MRI with an interval of 3–5 days. Volunteers received Gadobutrol in the first scan and Gd-DTPA in the second scan (group A) or vice versa (group B), which were determined randomly by SAS program (Version 9.2, SAS institute, Cary, NC, USA). All examinations were performed on a 1.5 tesla MR-scanner with a body coil containing at least 6 parallel channels (Siemens Avanto, Enlargen, Germany). A standard and comprehensive MR imaging protocol were performed, including pre- and post-T1-weighted image and MRA sequences. Three-dimensional T1-weighted images at the equilibrium phase (180 seconds) were acquired after injection. Detailed parameters are shown in Table 1.

Test boluses were 1.0 mL of Gd-DTPA and 0.5 mL of Gadobutrol, with an injection rate of 3.0 mL/seconds or 1.5 mL/seconds, respectively, followed by 30 ml of saline flush with the same injection rate. For enhancement, contrast materials were injected with 0.1 mmol/kg Gd-DTPA at a rate of 3.0 mL/seconds or 0.1 mmol/kg Gadobutrol at a rate of 1.5 mL/seconds, followed by 30 ml of saline flush with the same injection rate. All contrast materials were injected by a powerful injector (MEDRADINC, Indianola, USA). The investigators followed-up participants and documented any signs and symptoms within 72 hours of contrast agent administration.

### 2.3. Image Analysis

Two radiologists (12 years and 7 years' experience in abdominal imaging) read images on Syngo Imaging Workplaces (VersionVB35A, Siemens AG, Erlangen, Germany) independently, blinding to the contrast agents.

Abdominal vessels were divided into large, small, and minute blood vessels. Large vessels included celiac trunk, hepatic artery, the left gastric artery, splenic artery, proper hepatic artery, gastroduodenal artery, left hepatic artery, right hepatic artery, renal artery, the superior mesenteric artery, and the inferior mesenteric artery. Small vessels included the primary branches of the left and right hepatic arteries, primary branches of the left gastric arteries, primary branches of the renal arteries, and primary branches of the superior mesenteric arteries. Minute blood vessels included the second and third branches of the left and right hepatic arteries, second and third branches of the renal arteries, and second and third branches of the superior mesenteric arteries.

MRA analyses included qualitative analyses and quantitative analyses. Qualitative analyses for large and small vessels were performed by a three-point scale as follows [6, 12]: 0, not displayed; 1, displayed but not insufficient for diagnosis; and 2, displayed well for diagnosis. Qualitative analyses for minute small vessels were performed by a five-point scale as follows [13]: 0, not displayed; 1, displayed less than 25%; 2, displayed 25–50%; 3, displayed 50%–75%; 4, displayed more than 75%. The final scores of large, small and, minute small vessels were the score sum of relevant vessels, respectively.

MRA quantitative analyses were performed for large vessels. Signal intensity (SI) of vessels (SI_ves_) and SI of erector spinae (SI_mus_) were measured. The standard deviation of the background noise (SD_noi_) was measured in background region on the frequency-encoding direction. The corresponding sizes of regions of interest (ROI) for vessels were half of the diameter. The pixel sizes for SD_noi_ and SI_mus_ were both 50. The final values of SI and SD_noi_ were the average of two measurements. Signal-to-noise ratio (SNR) and contrast-to-noise ratio (CNR) were calculated as follows [14, 15]: SNR = SI_ves_/SD_noi_; CNR = (SI_ves_−SI_mus_)/SD_noi_.

For visceral organ enhancement analysis, pre-T1-weighted image (SI_pre_) and equilibrium phase images (SI_enh_) were analyzed. The ROIs (pixel size = 50) need to avoid vessel and lesions and were standardized for each organ: liver, on 2 different areas of the liver (left and right lobe) at the level of the hepatic hilum and right portal vein; spleen, in the parenchyma center at the level of the spleen hilum and spleen vein; pancreas, in the pancreatic body; and kidney, containing cortex and medulla at the level of the renal hilum and renal vein [16]. For each organ, a corresponding circular ROI was placed in the background region (pixel size = 50) on the frequency-encoding direction and defined as SD_noi_. The final value of SI and SD_noi_ were the average of two measurements. SNR and CNR were calculated with the following equations [16]: SNR = SI_enh_/SD_noi_; CNR = (SI_enh_−SI_pre_)/SD_noi_.

### 2.4. Statistical Analysis

A two-sided Wilcoxon signed-rank test was used to compare the qualitative results of vessels in Gadobutrol- and Gd-DTPA-enhanced MRA images. For assessment of quantitative parameters of large vessels and visceral organ enhancement in the intraindividual MR examinations with Gadobutrol and Gd-DTPA, the pairwise Wilcoxon signed-rank test was applied. A *p* value of <0.05 was established as statistically significant difference.

The interobserver agreement for qualitative analysis was assessed by Cohen's Kappa statistics [17]. Kappa values greater than 0.75 were taken to represent excellent agreement; values between 0.4 and 0.75 represented good agreement and values below 0.4 represented poor agreement. The interobserver agreement for quantitative analysis was assessed by Bland–Altman test. A *p* value of <0.05 was considered to be statistically significant.

Wilcoxon signed-rank test and Kappa statistics were performed using SPSS software (version 19, Chicago, IL, USA). Bland–Altman test was performed using MedCalc software (version 18.0, Ostend, Belgium).

## 3. Results

From December 2014 to December 2015, 40 volunteers (mean age 49.35 ± 8.67, with a range of 28–63 years old; 25 female and 15 male) in three medical centers were consecutively enrolled. Twenty volunteers received Gadobutrol in the first scan and Gd-DTPA in the second scan while the other 20 volunteers received GBCAs in opposite sequence. None of patients had adverse event.

### 3.1. CE-MRA Analyses

**Figure 1:**
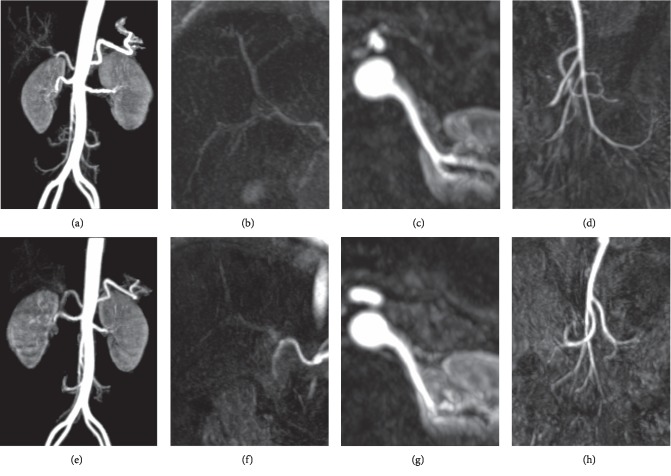
Contrast-enhanced magnetic resonance angiography (MRA) images of a 49-year-old man. Gadobutrol-enhanced MRA images: maximum intensity projection (MIP) image (a); multiplanner reformation (MPR) image of the hepatic artery (b); left kidney artery (c); the superior mesenteric artery (d). Gd-DTPA-enhanced MRA images: MIP image (e); MPR image of the hepatic artery (f); left kidney artery (g); the superior mesenteric artery (h). The quality analyses results for large, small, and minute vessels on Gadobutrol-enhanced MRA images are 22, 7, and 5 and those on Gd-DTPA-enhanced MRA images are 20, 6, and 4, respectively.

The qualitative analyses results for large vessels, small vessels, and minute small vessels of Gadobutrol and Gd-DTPA were 20.98 ± 2.11, 6.03 ± 1.03, and 3.41 ± 1.18 and 20.01 ± 2.18, 5.28 ± 1.67, and 2.61 ± 1.40, respectively (Table 2) (Figure 1). Wilcoxon signed-rank tests revealed Gadobutrol was significantly superior to Gd-DTPA for small vessels (*p*=0.028) and minute small vessels (*p*=0.007), with no statistical difference for large vessels (*p* > 0.05).

For quantitative analysis of large vessels, no statistic difference was found (*p* > 0.05).

### 3.2. CE-MRI of Visceral Organ Analyses

**Figure 2:**
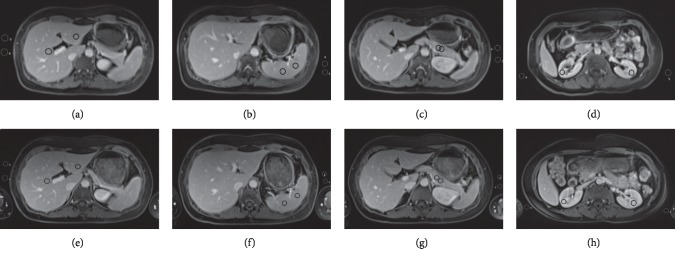
Abdominal visceral organ-enhanced images of a 56-year-old man. Equilibrium phase images of Gadobutrol-enhanced MRI: (a) liver (SNR 181.34, CNR 60.61); (b) spleen (SNR 202.06, CNR 109.39); (c) pancreas (SNR 161.11, CNR 54.65); (d) kidney (SNR 209.00, CNR 129.40). Equilibrium phase images of Gd-DTPA-enhanced MRI: (e) liver (SNR 145.56, CNR 44.48); (f) spleen (SNR 135.61, CNR 73.96); (g) pancreas (SNR 130.80, CNR 38.28); (h) kidney (SNR 187.62, CNR 101.84). The circles indicate ROIs.

The SNR and CNR of liver, spleen, pancreas, and renal tissues in the equilibrium phase of Gadobutrol-enhanced MRI and those of Gd-DTPA-enhanced MRI are shown in Table 3. Wilcoxon signed-rank tests revealed Gadobutrol-enhanced MRI with significant higher CNR of liver (*p*=0.003), spleen (*p*=0.001), and pancreas (*p*=0.001) and significant higher SNR of spleen (*p*=0.009) than those of Gd-DTPA-enhanced MRI, with no statistical difference for the others (*p* > 0.05) (Figure 2).

### 3.3. Agreement Analyses

The kappa values of two readers for qualitative analyses in large, small, and minute vessels were 0.909, 0.796, and 0.848, respectively. These indicated excellent agreement between two readers. Bland–Altman test of quantitative analyses showed the mean difference value of two readers was 19.41 (95% confidence interval, −13.01 to 51.82). The *p* value was 0.239, which indicated there was no statistical significance, and the consistency between the two readers was good.

## 4. Discussion

Gadobutrol and Gd-DTPA are two common GBCAs in clinic. Because of none literature has been retrieved on the applications of Gadobutrol and Gd-DTPA in abdominal CE-MRA and CE-MRI of a “one-stop” imaging, we innovatively focused our study on the “one-stop.” The research enrolled 40 consecutive volunteers. All volunteers underwent both Gadobutrol- and Gd-DTPA-enhanced MRA and MRI in a “one-stop.” All examinations were performed on the same MR-scanner with the same scan sequences and parameters in three medical centers. Results show Gadobutrol-enhanced MRA is superior to that of Gd-DTPA for small vessels and minute small vessels. Quantitative results of visceral organ enhancements on the equilibrium phase demonstrated Gadobutrol-enhanced MRI with higher enhancement than those of Gd-DTPA. We proved that Gadobutrol may be more suitable for abdominal one-stop examination for CE-MRA and CE-MRI than Gd-DTPA.

Previous research studies report that Gadobutrol-enhanced MRA could depict vascular wall as well as lumen. In 2003, Herborn et al. [6] reported Gadobutrol-enhanced MRA improved delineation of the pelvic arterial morphology compared with MRA performed with Gd-DTPA in five healthy volunteers. At the same time, a prospective, multicenter study including 182 patients presented that CE MRA with 1.0 mol Gadobutrol gave results comparable with those of digital subtraction angiography (DSA) for the larger arteries of pelvis and thigh in patients who had known or suspected disease of pelvic and peripheral arteries [18]. Goyen et al. [19] also report Gadobutrol shows advantages in total-body CE-MRA compared with that of Gd-DTPA. However, Fink et al. compared the signal characteristics and bolus dynamics of Gadobutrol and Gd-DTPA for CE-MRA of the upper torso, and they reported that Gadobutrol offered no relevant advantages [8]. One reason may be the scan technique. In Fink's study, consecutive MRA data were collected with a scan time of 43 seconds after injection, and the highest SNR of vessels were compared. Another explanation may be the injection rates, which were more quick than usual. Besides, they only compared large vessels. Except for these, the sample size is small with only ten volunteers, which may lead to deviation.

Recent study reported at equimolar doses of Gadobutrol demonstrated higher SNR and CNR than those of gadobenate dimeglumine on MRA images [20]. Our study proved Gadobutrol demonstrated higher SNR and CNR than Gd-DTPA in abdominal large vessels. However, the difference is with no statistic. In our study, quantitative analysis of CE-MRA was not applied for small vessels and minute small vessels, due to the thin diameter bringing about measurement bias. Considering Gadobutrol was superior to Gd-DTPA with a better delineation of small vessels and minute small vessels, we might suspect that Gadobutrol might have higher SNR and CNR than those of Gd-DTPA in small and minute small vessels in the abdomen. This speculation needs future confirmation.

Gadobutrol displayed well effective for CE-MRI of the body [21]. Our results indicate that abdominal visceral organs show higher enhancement in equilibrium phase images of Gadobutrol-enhanced MRI than those of Gd-DTPA at equimolar doses of gadolinium.

In spite this study was prospectively designed as an intraindividual comparison of two different contrast agents, there are some limitations. Firstly, the CE-MRA results lack comparison with DSA. Secondly, in spite our study was with the purpose of excluding anything other than contrast agent-induced effects at its best, noncontrast agent-related effects are inevitable and may affect the assessment. For example, differences in the location between the imaging coil due to physical positioning variations, movement and coil artifacts, variations in slice orientation etc., might exist. Thirdly, dynamic CE-MRI usually includes multiphase images; however, in our study, we could only acquire the equilibrium phase image for visceral organ enhancement, and SNR and CNR might not be always in accordance with diagnosis performance. In further, we would explore this application in patients such as with focal liver disease, liver transplantation patients, and so on.

## 5. Conclusions

This prospective intraindividual study innovatively attempted to compare Gadobutrol with Gd-DTPA at equimolar doses of gadolinium in abdominal CE-MRA and CE-MRI of a one-stop imaging. Results prove Gadobutrol may be more suitable for abdominal CE-MRA and CE-MRI. This research provides a reference for the clinical application and further evaluation in the clinic of Gadobutrol in abdominal CE-MRA and CE-MRI of a one-stop imaging.

## Figures and Tables

**Table 1:** Sequence parameters for MRA and MRI.

	Pre- and gadolinium-enhanced T1-weighted imaging	Pre- and gadolinium-enhanced MRA asset
Repetition time (ms)	4.74–5.41	2.94–3.02
Echo time (ms)	2.38	0.97–1.04
Flip angle (degree)	10	22–25
Bandwidth (Hz/pixel)	260	449
Acquisition matrix (pixel)	288 × 179,320 × 110	384 × 202,384 × 264
Field of view (mm^2^)	528 × 576,320 × 160	384 × 252,384 × 264
Slice thickness (mm)	2–2.8	1.2–1.4
Acquisition time (s)	18–22	19
No. of sections	72–96	80–88

MRA, magnetic resonance angiography; volumetric interpolated breath-hold examination is used for pre-T1-weighted imaging (WI) and gadolinium-enhanced T1WI; Fl3d1 is used for pre-MRA and enhanced MRA.

**Table 2:** Qualitative and quantitative analyses results of two contrast-enhanced MRA.

	Gadobutrol			Gd-DTPA			*p*
	Reader 1	Reader 2	Average	Reader 1	Reader 2	Average	Average
Qualitative analyses							
Large vessels	21.28 ± 2.22	20.68 ± 2.35	20.98 ± 2.21	20.08 ± 2.09	19.95 ± 2.43	20.01 ± 2.18	0.085
Small vessels	6.13 ± 1.16	5.93 ± 1.10	6.03 ± 1.03	5.08 ± 1.66	5.48 ± 1.83	5.28 ± 1.67	0.028
Minute blood vessels	3.45 ± 1.15	3.38 ± 1.28	3.41 ± 1.18	2.68 ± 1.47	2.55 ± 1.47	2.61 ± 1.40	0.007
Quantitative analyses for large vessels							
SNR	966.47 ± 318.18	994.07 ± 297.43	991.66 ± 318.11	907.18 ± 321.41	947.19 ± 305.39	927.18 ± 300.42	0.116
CNR	738.37 ± 327.26	829.22 ± 302.37	772.26 ± 316.16	796.47 ± 318.18	791.66 ± 318.11	795.32 ± 310.68	0.132

MRA, magnetic resonance angiography; SNR, signal-to-noise ratio; CNR, contrast-to-noise ratio.

**Table 3:** Quantitative analyses results of visceral organ enhancement of two contrast-enhanced MRI.

		Gd-DTPA			Gadobutrol			*p*
		Reader 1	Reader 2	Average	Reader 1	Reader 2	Average	Average
Liver	SNR	152.85 ± 43.48	137.07 ± 36.54	143.65 ± 37.05	150.73 ± 49.69	147.36 ± 37.28	147.52 ± 40.53	0.323
	CNR	44.50 ± 20.32	40.99 ± 15.79	42.32 ± 16.63	47.18 ± 20.53	50.84 ± 14.80	48.38 ± 16.54	0.003
Spleen	SNR	148.04 ± 44.62	144.43 ± 40.55	144.57 ± 38.07	147.75 ± 48.21	166.09 ± 36.88	154.83 ± 40.46	0.009
	CNR	77.90 ± 28.67	71.61 ± 27.71	73.87 ± 25.22	81.212 ± 31.14	90.39 ± 24.67	84.76 ± 26.80	0.001
Pancreas	SNR	156.74 ± 44.42	146.89 ± 33.05	150.72 ± 36.39	166.15 ± 51.06	152.75 ± 34.12	157.78 ± 39.33	0.056
	CNR	44.29 ± 19.48	39.29 ± 15.54	41.58 ± 16.08	50.05 ± 23.34	47.68 ± 15.48	48.26 ± 17.33	0.001
Kidney	SNR	187.13 ± 51.95	178.24 ± 41.84	181.02 ± 42.51	182.37 ± 38.81	185.41 ± 42.53	182.76 ± 37.35	0.628
	CNR	109.60 ± 39.92	106.60 ± 29.72	107.02 ± 32.08	106.76 ± 27.74	112.95 ± 30.46	109.31 ± 27.05	0.347

SNR, signal-to-noise ratio; CNR, contrast-to-noise ratio.

## Data Availability

The MR images and research data used to support the findings of this study were supplied by West China Hospital, Sichuan University, the First Hospital of Jilin University, and Sichuan Cancer Hospital and so cannot be made freely available. Requests for access to these data should be made to Xijiao Liu, e-mail: bless_jiao@163.com; telephone: +86–15928745612; fax: +86-28-85423680(O); address: Department of Radiology, West China Hospital, Sichuan University, Chengdu, Sichuan province, China (postcode: 610041).
